# Silicon-Containing π-Conjugated Schiff Base Oligomers with Naphthalene or Binaphthalene Moieties in the Backbone: Synthesis and Study of Properties

**DOI:** 10.3390/polym17101316

**Published:** 2025-05-12

**Authors:** Enzo González, Alexis F. González, Andrea P. Mariman, Camilo I. Jara, Joel D. Velázquez, César Saldías, Eduardo Schott, Ximena Zarate, Alain Tundidor-Camba, Patricio A. Sobarzo, Claudio A. Terraza

**Affiliations:** 1 Research Laboratory for Organic Polymers, Faculty of Chemistry and of Pharmacy, Pontificia Universidad Católica de Chile, P.O. Box. 306, Post 22, Santiago PC. 7820436, Chile; enzo.gonzalez@uc.cl; 2 Department of Physical Chemistry, Pontificia Universidad Católica de Chile, Santiago PC. 7820436, Chilecasaldia@uc.cl (C.S.); 3 Instituto de Ciencias Químicas, Facultad de Ciencias, Universidad Austral de Chile, Valdivia 5090000, Chilejoel.velasquez@uach.cl (J.D.V.); 4 Department of Inorganic Chemistry, Pontificia Universidad Católica de Chile, Santiago PC. 7820436, Chile; adschott@uc.cl; 5 Instituto de Ciencias Químicas Aplicadas, Facultad de Ingeniería, Universidad Autónoma de Chile, Santiago CP. 4780319, Chile; ximena.zarate@uautonoma.cl; 6 Department of Chemical & Biological Engineering, The University of Alabama, P.O. Box 870203, Tuscaloosa, AL 35487-0203, USA; atundodorcamba@ua.edu; 7 Departamento de Polímeros, Facultad de Ciencias Químicas, Universidad de Concepción, Edmundo Larenas 129, Casilla 160-C, Concepción CP. 4030000, Chile

**Keywords:** silane-based materials, Schiff base oligomers, tetraphenylsilane core, naphthalene, TD-DFT calculations

## Abstract

Four silane-containing Schiff base oligomers (o-SBNs and o-SBBs) were synthesized by high-temperature polycondensation reactions using silicon-based dialdehydes with naphthalene and 1,1’-binaphthalene diamine derivates. The samples showed a moderate solubility in common organic solvents, where the incorporation of TPS cores into o-SBN2 allows the formation of highly soluble material in non-polar solvents with higher molecular weights (11.58 kDa) and polydispersity. All oligo-SBs displayed high thermal resistance (above 450 °C), showing enhanced thermal stability for TPS-containing oligomers, with the degradation temperature exceeding 530 °C (o-SBB2) and high T_g_ values due to the higher aromatic content granted by TPS and 1,1’-binaphthalene moieties. Optical results of the oligo-SBs showed broad absorption and emission behavior in the visible spectrum, ranging from deep blue (o-SBN1 and o-SBB1) to blue (o-SBN2 and o-SBB2). The structure promotes a clear bathochromic shift for TPS-based oligomers, attributed to an extended π-conjugation across the backbone. In addition, the π-π overlap effect highlights larger Stokes shifts for the DMS core oligomers o-SN2 (133 nm) and o-SBB1 (195 nm). The oligo-SBs were found to be wide-bandgap materials, with E_g_^opt^ values in the range of 2.60 eV to 3.67 eV. The higher molecular weight of o-SBN2, which provided an extended π-conjugation, allows the lowest value of E_g_^opt^ (2.60 eV) to be achieved. In addition, DFT, TDDFT and EDDM calculations were performed on trimeric oligo-SBs, revealing that HOMOs are localized in the amine-terminal fraction, while LUMOs are localized over the terminal aldehyde groups. These findings highlight the used DMS and TPS cores in Schiff base materials, providing valuable insights into fine-tuning physicochemical properties through the use of suitable building blocks and their potential as optoelectronic materials.

## 1. Introduction

Highly π-conjugated materials have been extensively studied by many research groups over last few decades due to their great versatility and potential applications in different fields such as industry, energy, medicine and daily life [1]. In this sense, π-conjugated polymers have become key components in the development of new technologies and advances in the field of organic optoelectronics, used as a primary or complementary layer in polymer solar cells (PSCs), polymer light-emitting diodes (PLEDs) and energy storage devices [1,2,3]. Their remarkable physicochemical properties, such as broad absorption/emission behavior and tunable energy levels, are extrinsic to π-conjugated polymers. In addition, their lightweight nature, large-area processability, flexibility and low cost make them highly attractive for technological advances [1,4,5].

Schiff base polymers (SBps) have been highlighted among the π-conjugated materials for their ubiquitous optoelectronic properties and high thermomechanical stability, having been studied in different technological applications such as solar energy, light-emitting devices, organic transistors, catalysis and energy prototypes [6,7,8,9,10,11]. The imine linkages (HC=N) are characteristic of SBps and can be easily synthesized from diamine and dialdehyde monomers, allowing the formation of a wide range of chemical architectures, making them a versatile type of π-conjugated materials. Thus, the combination of physicochemical characteristics and easy access to multiple chemical architectures highlights the potential of these type of materials for applications and further integration into applied technologies [9,12,13]. In this regard, properly designed single-molecule Schiff bases or monomers for SBps allow their use as building blocks for hole transport materials in perovskite solar cells, where long-term stability is enhanced [8,14,15]. Similarly, SBps can be used as active materials in solid-state emitting devices with high quantum yield or polymeric Schiff base networks that could emit bright near-white fluorescence [16,17,18]. Furthermore, SBps containing suitable building block units can be used as anode materials for energy storage and as covalent organic polymer material coated nickel foam electrodes with high cyclic stability for supercapacitor application [19].

π-conjugated materials containing group IV-A atoms as central core structures have long been used to modulate optoelectronic properties as well as to improve long-term thermal and morphological stability [20,21]. Recent studies have demonstrated the successful integration of silicon-containing π-conjugated materials into advanced devices such as electrochromic coatings, photodetectors and energy storage systems, highlighting their potential for multifunctional optoelectronic applications [22,23,24]. Moreover, their tunable properties and thermal resilience make them promising candidates for next-generation technologies requiring operational robustness and adaptive functionality. In this sense, silane-based materials with dimethyl diphenylsilane (DMS) and tetraphenylsilane (TPS) moieties have attracted attention as useful conjugation-breaking spacer building blocks for wide-bandgap optoelectronic materials with high thermal stability in light-emitting applications [20,25,26,27]. Hence, innovative applications have emerged, such as electrochromic polymers for adaptive camouflage devices based on TPS core polymers, where the incorporation of thiophene-based units as well-defined redox linkages enables ion storage layers in a dual-polymer electrochromic device [28]. Moreover, the incorporation of TPS cores with a “twisted” geometry leads to highly soluble small molecules or polymeric backbones, yielding amorphous solid-state films suitable for large-area processing and low-cost fabrication techniques [21,29,30].

Proper design of the chemical building blocks for wide-bandgap materials with high thermal stability is a crucial step in identifying potential candidates for optoelectronics. Based on the above considerations on Schiff bases and silicon-containing π-conjugated materials, our research group has been devoted to the synthesis, characterization and physicochemical study of highly conjugated silane-based Schiff base oligomers (oligo-SBs) and polymers with DMS and TPS core backbones. Therefore, this work reports the successful synthesis of four new π-conjugated oligo-SBs (o-SBNs and o-SBBs) incorporating a DMS/TPS core conjugation-breaking spacer, in combination with naphthalene and 1,1’-binaphthalene moieties in the backbone.

## 2. Materials and Methods

### 2.1. Materials

n-butyllithium 2M in cyclohexane, anhydrous calcium sulfate, 1,4-diaminonaphthalene (dAN), 1,4-dibromobenzene, dichlorodimethylsilane, dichlorodiphenylsilane, anhydrous N,N-dimethylacetamide (DMAc), 1-naphthylamine, p-toluensulfonic acid monohydrate (TsOH) and tin(II) chloride dihydrate were acquired from Sigma-Aldrich (St. Louis, MO, USA). Sodium nitrite and sodium sulfite were acquired from JT Baker (Nueva Jersey, USA). Dichloromethane (DCM), 1,4-dioxane, dimethyl sulfoxide (DMSO), ethyl ether, n-hexane, magnesium sulphate and sodium acetate were obtained from Merck (Darmstadt, Germany). 4-formylphenylboronic acid and bis(triphenylphosphine)palladium(II) dichloride were purchased from AK Scientific (Union City, USA). All solvents and reagents were used as received without further purification.

### 2.2. Synthesis of Precursors and Monomers

The synthetic route for the precursors and monomers is shown in Scheme S1. The precursor (*E*)-1,2-di(naphthalen-1-yl)diazene (AzDN) was synthesized in two stages based on Lange’s method [31]. Then, the monomer [1,1’-binaphthalene]-4,4’-diamine (dABN) was prepared by reduction of AzBN using tin(IV) chloride in methanol [31]. Additionally, silicon-containing monomers (M1 and M2) were synthesized from their respective silane-based precursors (P1 and P2) through Suzuki–Miyaura cross-coupling reactions, using bis(triphenylphosphine)palladium(II) dichloride and 4-formylphenylboronic acid, according to the previously reported literature [32,33]. The structure of the precursors and monomers was confirmed by spectroscopic techniques and elemental analysis.

### 2.3. Synthesis of Oligo-SBs

**Figure 1:**
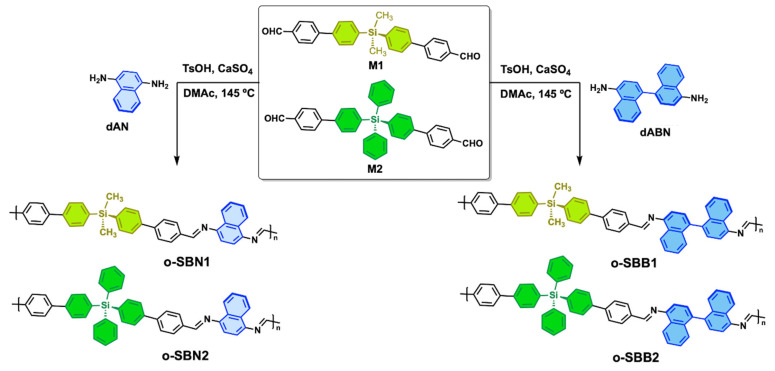
Synthetic route of the o-SBNs and o-SBBs series.

All oligomers were obtained following previous reports by high-temperature polycondensation reactions under a nitrogen atmosphere (Figure 1) [8,34]. In a typical experiment, using o-SBB1 as an example, silane-based monomer M2 (1 mmol), TsOH (43 mg, 0.32 mmol) and anhydrous CaSO_4_ (65 mg, 0.48 mmol) were dissolved in anhydrous DMAc (5 mL) and heated at 65 °C for 15 min. Then, a solution of dAN (1 mmol in 5 mL of anhydrous DMAc) was slowly added, stirred for 1.5 h, and then the temperature was increased to 145 °C and maintained for 20 h. After this time, the reaction mixture was cooled, poured into water (100 mL) and stirred for 3 h at room temperature. The precipitate was filtered under a vacuum, washed extensively with water and then with methanol in a Soxhlet apparatus for 48 h, followed by drying in vacuum oven at 50 °C for 20 h. The oligomers were obtained as yellow to dark orange solids.

o-SBN1: Yield: 84%. IR-TF (KBr pellets) cm^−1^: 3061, 3013 ν(H-C, arom.), 2959, 2944 ν(H-C, aliph.), 2892, 2776 ν(CO-H, term. ald.), 1690 ν(C=O, term. ald.), 1622 ν(CH=N), 1590, 1558 ν(C=C, arom.), 1378 ν(C-N), 1296, 1242, 1100 ν(Si-C), 810 γ(*p*-subst.) and 760 γ(*mono*-subst.). Anal. Elem. Calcd. para C_38_H_30_N_2_Si (542.76 g/mol)_n_: C, 84.09%; H, 5.57%; N, 5.16%. Found: C, 81.24%; H, 5.53%; N, 4.45%.

o-SBN2: Yield: 87%. IR-TF (KBr pellets) cm^−1^: 3372 ν(N-H, term. amine), 3060, 3035, 3010 ν(H-C, arom.), 2837, 2770 ν(CO-H, term. ald.), 1692 ν(C=O, term. ald.), 1620 ν(CH=N), 15γ94, 1557, 1477 ν(C=C, arom.), 1374 ν(C-N), 1290, 1190, 1102 ν(Si-C), 815 γ(*p*-subst.), 728 γ(*mono*-subst.) and 693 γ(Si-C, arom.). ^1^H NMR (400 MHz, CDCl_3_) δ ppm: 10.06 (CHO, term.), 8.65 (HC=N), 8.50–7.30 (H, arom.) and 5.29 (NH_2_, term.). Anal. Elem. Calcd. para C_48_H_34_N_2_Si (666.90 g/mol)_n_: C, 86.45%; H, 5.14%; N, 4.20%. Found: C, 82.98%; H, 5.09%; N, 3.82%.

o-SBB1: Yield: 81%. IR-TF (KBr pellets) cm^−1^: 3053, 3012 ν(H-C, arom.), 2940, 2913 ν(H-C, aliph.), 2835, 2720 ν(CO-H, term. ald.), 1691 ν(CH=O, term. ald.), 1620 ν(C=N), 1598, 1550 ν(C=C, arom.), 1372 ν(C-N), 1240, 1108 ν(Si-C), 810 γ(*p*-subst.) and 760 γ(*mono*-subst.). Anal. Elem. Calcd. para C_48_H_36_N_2_Si (668.92 g/mol)_n_: C, 86.19%; H, 5.42%; N, 4.19%. Found: C, 84.49%; H, 5.56%; N, 3.90%.

o-SBB2: Yield: 91%. IR-TF (KBr pellets) cm^−1^: 3434, 3428 ν(N-H, term. amine), 3065, 3039, 3010 ν(H-C, arom.), 2858, 2741 ν(CO-H, term. ald.), 1696 ν(C=O, term. ald.), 1619 ν(CH=N), 1590, 1560, 1418 ν(C=C, arom.), 1384 ν(C-N), 1279, 1103 ν(Si-C), 812 γ(*p*-subst.), 760, 724 γ(*mono*-subst.) and 699 γ(Si-C, arom.). ^1^H NMR (400 MHz, CDCl_3_) δ ppm: 10.07 (CHO, term. ald.), 8.73 (HC=N), 8.60–7.35 (H, arom.) and 5.30 (NH_2_, term.). Anal. Elem. Calcd. para C_58_H_40_N_2_Si (793.06 g/mol)_n_: C, 87.84%; H, 5.08%; N, 3.53%. Found: C, 85.16%; H, 5.19%; N, 3.28%.

### 2.4. Measurements

FT-IR spectra were acquired on a JASCO FT-IR 4200 Spectrometer (Jasco, Maryland, USA) using KBr pellets. ^1^H NMR spectra were obtained in solution on a Bruker Avance III HD-400 (Bruker, Karlsruhe, Germany) using acetone-*d6* or chloroform-*d* as a solvent with tetramethylsilane (TMS) as an internal standard. Elemental analyses were performed on a LECO CHNS-932 Elemental Analyzer (Leco, Michigan, USA). Thermal studies were performed on a Mettler Toledo TGA/SDTA 851 Thermogravimetric Analyzer (TGA) (Mettler Toledo, Columbus, Ohio, USA) with a scan rate of 10 °C/min in a nitrogen atmosphere. Differential scanning calorimetry (DSC) was performed on a Mettler Toledo DSC821 (Mettler Toledo, Columbus, Ohio, USA) at heating and cooling rates of 10 °C/min and 30 °C/min, respectively. Molecular weight determination was performed by gel permeation chromatography (GPC) on a Wyatt Technology Dawn EOS HPLC instrument equipped with an Optilab DSP differential refractive index detector (Wyatt Technology, Santa Barbara, CA, USA). Samples were dissolved in THF (soluble fraction) and filtered prior to measurement using a 0.45 µm nylon membrane. Elution was performed at a flow rate of 1.0 mL/min at 25 °C, using polystyrene standards for the calibration curve. UV–vis and fluorescence spectroscopy studies were carried out in solution (soluble fraction) and performed on a Perkin-Elmer Lambda 35UV/VIS Spectrometer (Perkin-Elmer, Waltham, MA, USA) and a Jasco FP-6200 Spectrofluorometer (Jasco, MD, USA), respectively.

### 2.5. DFT Simulations

All computational calculations were performed using the Gaussian 09 computational package [35]. The studied structures were optimized in their ground state. These calculations were carried out using Becke’s three-parameter nonlocal hybrid exchange functional and the Lee–Yang–Parr nonlocal correlation functional, including the CAM-B3LYP long-range interaction correction [36]. The 6-311G* basis set was used for C, S, N and H atoms [37]. The Hessian was also calculated for all derivatives to ensure that the obtained structure corresponds to a local minimum, confirming that no imaginary frequencies were presented. In all cases, chains with three repeating units were considered, which accurately describe the experimental results. In addition, time-dependent DFT (TDDFT) calculations were performed using the same theory level to simulate the oligomeric UV–vis spectra. Thus, first-principles calculations were employed to obtain accurate excitation energies and oscillator strengths related to the molar extinction coefficient. The polarizable continuum model (PCM) using DCM as solvent was applied for geometry optimization and TDDFT calculations to include solvent effects [38].

## 3. Results and Discussion

### 3.1. Synthesis and Characterization of Monomers

M1 and M2 were obtained from the respective dibromide derivatives with DMS- and TPS cores by Suzuki–Miyaura cross-coupling reactions, using palladium(II) catalyst and 4-formylphenylboronic acid, with yields of 56% and 61%, respectively (Figure S1) [32,33]. The NMR spectra of these monomers are shown in Figures S6 and S7. dABN was synthesized according to the procedure of Cohen et al. using naphthalen-1-amine, which was transformed into its respective azo-precursor (AzDN) via the Van Slyke diazo self-coupling reaction, followed by reduction with tin(II) salt and subsequent acid-catalyzed benzidine rearrangement to obtain the diamine monomer with a 29% overall yield (Figure S1) [31]. Structural characterization of these compounds was performed by FT-IR, NMR and elemental analysis techniques.

### 3.2. Synthesis and Characterization of Oligo-SBs

Four oligomers were obtained through a high-temperature polycondensation method using TsOH as an acid catalyst (pH 5.0) and drying agent (Figure 1). The one-naphthalene series (o-SBNs) was achieved using a commercial naphthalene-1,4-diamine (dAN) with silane-based dialdehydes (M1 and M2). Similarly, the binaphthalene-based series (o-SBBs) was synthetized from dABN [8,16,39]. All oligomers were purified by Soxhlet extraction using methanol as a solvent to remove monomers traces, and, after a thorough drying process, a colored solid was obtained. The results of elemental analysis showed that the C/H and C/N ratios agree with the empirical formulas of the repeating units.

**Figure 2:**
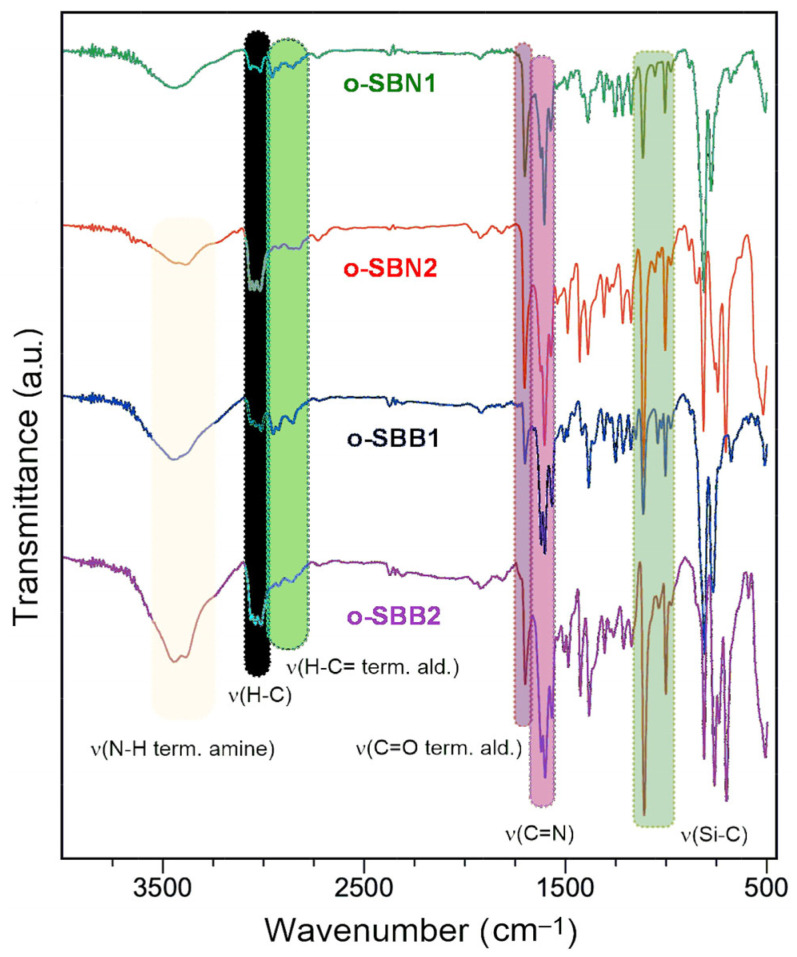
FT-IR spectra of o-SBNs and o-SBBs in KBr pellets.

The FT-IR spectra (Figure 2) show the characteristic band of the imine bond between 1622 and 1619 cm^−1^, as well as bands centered at 1370 cm^−1^ attributed to ν(C-N) stretching. The DMS core for o-SBN1 and o-SBB1 was observed between 1254 and 1130 cm^−1^, while the TPS core stretching bands for o-SBN2 and o-SBB2 were evidenced in the range of 1218–1110 cm^−1^. Moreover, the presence of DMS cores was confirmed through ν(C-H) stretching of the methyl groups, centered at 2934 cm^−1^, while ν(C-H) stretching of phenyl rings of the TPS core was found at ca. 3034 cm^−1^ [40]. The oligomeric nature of the samples was consistent with signals associated with the terminal aldehyde groups, such as stretching bands between 2892 and 2720 cm^−1^ for ν(CO-H) and the carbonyl bond, centered at 1693 cm^−1^ for ν(C=O), similar to previous work [39,41]. Furthermore, in the case of TPS core, an additional signal for terminal amino linkages ν(N-H, term. amine) was observed at 3372 cm^−1^ for o-SBN2 and in the range of 3434–3428 cm^−1^ for o-SBB2. Based on the FT-IR spectra and elemental analysis results, it is possible to highlight the oligomeric nature of the samples, similar to previous silicon-based oligo-SBs containing DMS and TPS core in the backbone [13,39,41].

**Figure 3:**
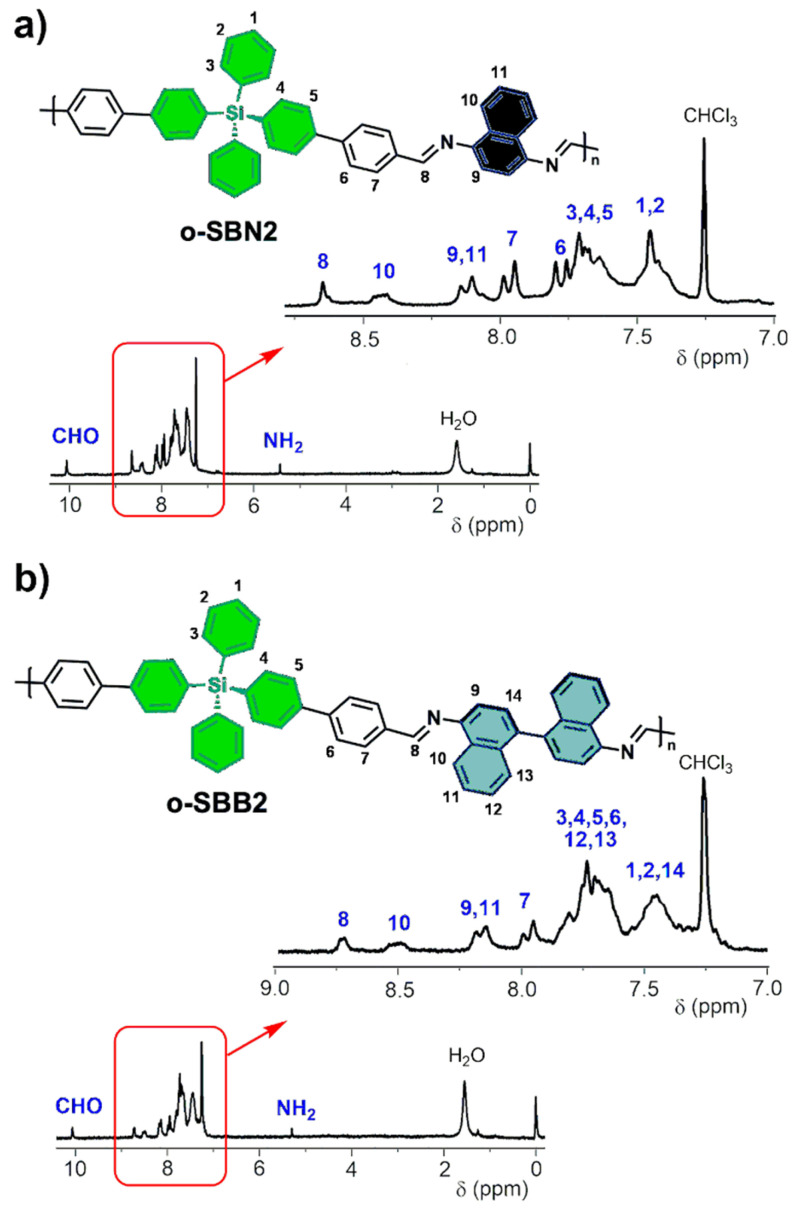
Full ^1^H NMR spectra (CDCl_3_) and expanded aromatic region of (**a**) o-SBN2 and (**b**) o-SBB2.

Due to the low solubility of the samples containing DMS cores in deuterated solvents, ^1^H NMR analysis was carried out only for the oligomers with TPS cores (Figure 3). In the spectra of both samples, signals corresponding to the aromatic hydrogens of byphenyl moieties afforded by dialdehyde monomers and naphthalene or 1,1’-binaphthalene units were detected at 8.70 ppm and 7.25 ppm. In addition, imine protons were assigned at signals at 8.65 ppm (o-SBN2) and 8.73 ppm (o-SBB2).

For the protons of the naphthalene and 1,1’-binaphthalene units, signals were observed in lower fields compared to reported oligo-SBs with similar structure [13]. In agreement with other Schiff base oligomers and polymers, the spectra of both oligo-SBs displayed signals corresponding to the protons of terminal aldehyde groups at ca. 10.06 ppm, as well as the terminal amino signals centered at 5.30 ppm. These findings provide further evidence for the oligomeric nature of these new silicon-based materials [41].

### 3.3. Solubility, Molecular Weights and Thermal Properties of Oligo-SBs

The solubility of the oligo-SBs in common organic solvents was investigated, and the results are summarized in Table S1. All samples were found to be insoluble in polar protic solvents (C_1–4_ alcohols), similar to the silicon-based oligo-SBs and SB-polymers previously reported [13,39,41]. The results indicate that the samples only reach partial solubility in high-boiling point aprotic polar solvents such as chlorobenzene, DMF and DMSO, even under heat application. Despite the low solubility in most solvents tested, oligo-SBs with TPS cores (o-SBN2 and o-SBB2) showed higher solubility in solvents such as chloroform and THF. o-SBN2 exhibited the best solubility among the oligo-SBs, forming dark-yellow to pale-yellow solutions.

The differences in the solubility behavior of the oligomers can be explained by considering both the geometry of the silane core (DMS and TPS units) and the inter- and intra-chain interactions. The presence of imine linkages increases the rigidity of the chains, enhancing the π-π stacking interactions between oligomer chains. In the case o-SBN2 and o-SBB2, the higher hydrodynamic volume induced by the “twisted” geometry of the TPS core relative to the DMS core reduces the packing forces, which facilitates the solvent penetration into the oligomeric matrix, thus improving the solubility of the samples. For the naphthalene-containing oligomer (o-SBN2), the lower rigidity due to the reduced aromatic content minimizes inter- and intra-molecular stacking. In contrast, for 1,1’-binaphthalene-containing oligomers (o-SBB1 and o-SBB2), the stronger π-π interactions derived from their extended aromatic system tend to reduce the solubility. In o-SBB1 containing a DMS core, the combined effect of a higher aromatic content and an efficient packing group contributes to reducing its solubility. The observed solubility trend (o-SBN2 > o-SBB2 > o-SBN1 > o-SBB1) highlights the critical balance between structural rigidity and aromatic content in the solubility behavior of these oligo-SBs.

The molecular weights (M_w_ and M_n_), polydispersity index (PDI) and degree of polymerization (DP) of the oligo-SBs were determined from the THF-soluble fractions by GPC experiments (Figure S2), with the exception of o-SBB1 due to its low solubility. These results are summarized in Table 1. Oligomers with bulky cores and “twisted” geometry (o-SBN2 and o-SBB2) have the highest M_w_ values (10–11.5 kDa). This behavior can be attributed to the higher hydrodynamic volume, which effectively reduces chain packing and promotes oligomer growth during the polycondensation reaction. In contrast, o-SBN1 containing a DMS core showed the lowest M_w_ value (5.10 kDa) due to the lower solubility of the growing chains.

The PDI values (M_w_/M_n_) reveal the influence of the structural design of the monomers and the complexity of the polycondensation reaction. The large variability in chain lengths shown by o-SBB2 (PDI = 2.21) with respect to the o-SBN series oligomers, in particular with o-SBB1 (PDI = 1.75), can be attributed to the presence of the 1,1’-binaphthalene moiety. This structural element modulates the solubility of the sample and thus its growth during the polycondensation reaction. The aforementioned π-stacking interactions between chains restrict the mobility of the oligomer backbone, favoring the variability of the PDI. The values of the degree of polymerization (M_n_/molecular weight of the repeating unit) show that on average, o-SBN2 is composed of 10 repeating units (decamer), o-SBN1 corresponds to a pentamer, while o-SBB2 would be a hexamer. These results show that the nature of the diamine monomer and the silane core are elements that can be used to modulate these parameters, establishing a relationship between the molecular structure and the macroscopic properties of the oligo-SBs.

**Figure 4:**
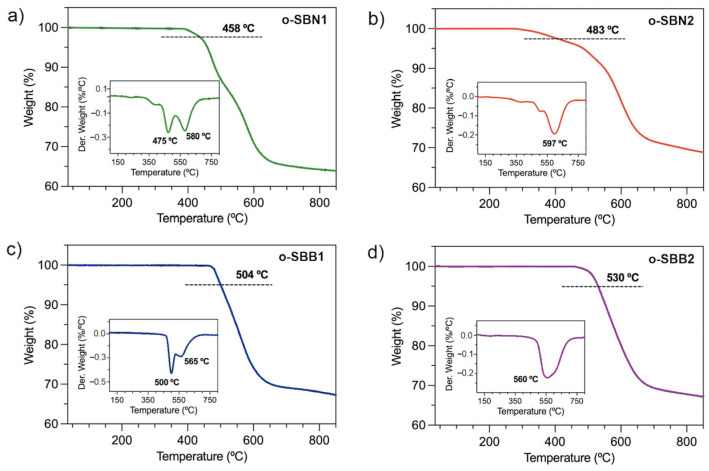
Thermograms of oligo-SBs in a nitrogen atmosphere, where the inset figures represent the DTGA curves. (**a**) o-SBN1, (**b**) o-SBN2, (**c**) o-SBB1 and (**d**) o-SBB2.

The thermal properties of the oligo-SBs were evaluated through TGA and DSC experiments under a nitrogen atmosphere. The results are summarized in Table 1, and the curves are shown in Figure 4 and Figure S3, respectively.

The thermograms show the 5% weight loss used as an indicator parameter, displaying high thermal stability for these oligo-SBs [21,34,42]. Thus, for oligomers containing naphthalene units in their backbone (o-SBN1 and o-SBN2), the 5% weight loss was detected at 458 °C and 483 °C, respectively. In contrast, this parameter is 50 °C higher for the series containing 1,1’-binaphthalene moieties, reaching 504 °C for o-SBB1 and 530 °C for o-SBB2. Moreover, at a 10% weight loss, the thermal stability of o-SBNs and o-SBBs was observed above 480 °C, highlighting the remarkable thermal stability of these new Schiff base materials [7,43,44].

The DTGA curves (Figure 4, inset) allow us to identify key differences in the thermal stability and degradation processes of the oligomers as a function of their aromatic content and the nature of the silane-based core. Oligomers containing TPS cores exhibit a single decomposition stage at temperatures close to 600 °C, suggesting that higher aromatic content of this moiety compared to the DMS core significantly improves the thermal resistance of the samples. For the DMS-containing series, the first decomposition process occurs at temperatures between 475 and 500 °C, which could be associated with the cleavage of the imine bond and decomposition of the naphthalene system. The second thermal event was observed at temperatures between 565 and 580 °C, which would correspond to the complete degradation of the oligomeric chains. The residual mass at 850 °C (R) for the silicon-based oligo-SBs was found to be similar, with slightly higher values for o-SBN2 and o-SBB2 compared to o-SBN1 and o-SBB1, highlighting the superior thermal stability of the TPS-containing structures versus the DMS-based oligo-SBs.

The results of DSC experiments (Figure S3) showed that the glass transition temperature (T_g_) values follow a clear trend: o-SBN1 < o-SBN2 < o-SBB1 < o-SBB2, where the increase in aromatic content, provided by the diamine monomer and the presence of TPS cores, significantly enhances the T_g_ values. Thus, the TPS core samples showed T_g_ values 40 °C higher than those of their DMS core counterparts, while the use of the 1,1’-binaphthalene unit instead of the naphthalene unit increases this parameter above 210 °C. This analysis shows that the stiffness of the diamine moiety is the determining factor in the increase in T_g_ values in the oligomers, probably due to the increase in the interchain packing forces.

### 3.4. Optical and Electronic Properties of Oligo-SBs

**Figure 5:**
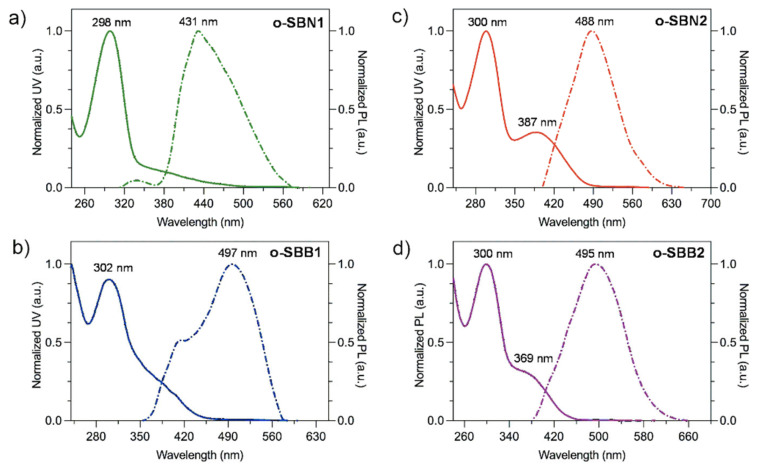
UV–vis (solid line) and PL (dashed line) spectra of o-SBNs and o-SBBs series in CHCl_3_-diluted solutions. (**a**) **o-SBN1**, (**b**) **o-SBN2**, (**c**) **o-SBB1** and (**d**) **o-SBB2**.

The optical and electronic properties of the silicon-based oligo-SBs were studied using UV–vis and photoluminescence spectroscopy. For this purpose, all the samples were treated with chloroform, and the resulting soluble fractions were used for optical measurements. The spectra are shown in Figure 5, and the results are summarized in Table 2.

In the UV–vis spectra (Figure 5), the absorption bands for the o-SBNs and o-SBBs oligomers were observed in the range of 298 nm to 387 nm. In the case of the DMS-containing series, a single absorption band was observed near 300 nm, which was attributed to π-π* and n-π* electronic transitions of the aromatic system of benzene rings and naphthalene moieties, as well as imine linkages [7,41]. For the oligomers containing TPS cores, the UV–vis spectra displayed two well-defined absorption bands: the first at 300 nm and the second band at 387 nm for o-SBN2 and at 369 nm for o-SBB2. The higher-energy band (300 nm) could be associated with π-π* transitions in both the benzene rings of the naphthalene or 1,1’-binaphthalene moieties and the benzene linked to TPS cores provided by dialdehyde monomers. Likewise, bands at lower energies were associated with n-π* electronic transitions of imine linkages along the oligomeric chains [34,45,46].

The trend observed by lower-energy absorption bands (λ_max_), follows the order o-SBN2 > o-SBB2 > o-SBB1 > o-SN1 (Table 2). The presence of TPS cores in the o-SN2 and o-SBB2 oligomers results in higher aromatic content and more efficient π-conjugation through the benzene-TPS-benzene-imino-naphthalene/1,1’-binaphthalene segments.

The structural design of the oligomers reveals a bathochromic shift in the absorption bands, which could be correlated with the aromatic content and building block units present in the repeating unit. In the o-SBNs series, the incorporation of TPS cores induces a shift of 89 nm for o-SBN2 compared to o-SBN1 with DMS cores. Likewise, the o-SBBs oligomers showed this effect with a smaller shift (67 nm). In this sense, in oligomers with TPS cores, the bathochromic shift is related to greater π-conjugation, whereas oligomers with DMS cores (o-SBN1 and o-SBB1) show a smaller effect. This behavior could be attributed to the higher aromatic content in the repeating units containing a TPS core, promoting a better π-overlap between aromatic systems (benzene, naphthalene and 1,1′-binaphthalene) and a higher π-electron delocalization along the chains, similar to previously reported oligo- and poly(azomethine)s [41,47,48]. Similarly, in oligomers with 1,1′-binaphthalene units (o-SBBs), the bathochromic shift could be enhanced by the greater coplanarity of these units in the backbone, contributing to a more effective π-conjugation through the imino groups. Although o-SBB2 has a higher aromatic content in its repeating unit compared to o-SBN2, its higher λ_max_ could be attributed to its higher molecular weight and better π-overlap in longer chains.

The photoluminescence (PL) spectra of the oligo-SBs (Figure 5, dashed line) were obtained using the lowest-energy absorption band (λ_max_) for each oligomer as the excitation wavelength, and the resulting λ_max_^em^ are summarized in the Table 2.

All oligomers exhibited remarkable fluorescence with emission bands ranging from 431 nm to 497 nm. For the o-SBN series, a 57 nm bathochromic shift was observed for o-SBN2 (λ_max_^em^ = 488 nm) compared to o-SBN1, which showed a λ_max_^em^ of 431 nm. In contrast, in oligomers containing 1,1’-binaphthalene moieties (o-SBBs), a slightly opposite effect was observed, where o-SBB1 exhibited a λ_max_^em^ of 497 nm compared to o-SBB2 (λ_max_^em^ = 495 nm), defining a hypsochromic effect. For all oligo-SBs, emission was observed in the blue–violet region of the visible spectrum, which could be attributed to a more extended π-conjugation of TPS-based oligomers (o-SBN2 and o-SBBs), which could stabilize the singlet excited state due to higher electron delocalization, similar to previously reported oligo-SBs [49,50,51,52].

All oligo-SBs exhibited large Stokes shift values (Table 2), between 100 nm and 200 nm, revealing significant differences in their structural design. In the o-SBN series, the Stokes shift increased 32 nm when the DMS core was replaced by the TPS core, whereas in the o-SBB series, this structural modification triggered a more pronounced shift (69 nm), highlighting the electronic effect of the TPS core. The systematically higher Stokes shift values recorded in the o-SBB series are influenced by the 1,1’-binaphthalene moieties, where the coplanarity facilitates π-conjugation in the excited state while increasing intermolecular interactions, resulting in a slightly hypsochromic shift. The smaller Stokes shift for o-SBB2 compared to o-SBB1 could be indicative that TPS cores not only affect the π-conjugation but also the electronic density distribution in the excited state, generating a blue shift compared to using DMS cores as building blocks.

These highly aromatic oligo-SBs, with their large Stokes shifts, could be promising candidates for optoelectronic applications where the minimization of self-absorption and light-scattering processes are essential parameters for high-performance materials, thus optimizing the purity and efficiency of light emission [26,53]. These results suggest that structural relaxation in the excited state, enhanced by the flexibility of the DMS cores, contributes to the large Stokes shifts observed in the o-SBs series.

From the UV–vis spectroscopy results, the optical bandgap (E_g_^opt^) of the oligomers was determined using the absorption onset (λ_onset_) of the lowest-energy band. This calculation was performed using the Tauc equation (Equation (1)) and its approximation for the study of conjugated materials (Equation (2)) [54,55,56].

*A* = *B* (*hν* − *E_g_*)^1/2^/*hν* (1)

E_g_^opt^ = 1241/λ_onset_ (2)

where *A* corresponds to the absorbance of the sample, *B* is the material constant, *h* is Planck’s constant and *ν* corresponds to the frequency. Thus, assuming that for conjugated materials *A* = 0 and *h*ν = E_g_ in Equation (1), the approximate expression can be used to reliably estimate the optical bandgap (E_g_^opt^) [57]. The Eg^opt^ values of the oligo-SBs follow the trend o-SBN1 > o-SBB1 > o-SBB2 > o-SBN2, ranging from 3.67 eV to 2.60 eV. In the o-SBN series, a significant decrease in this parameter (1.07 eV) was observed when the DMS core was replaced by a TPS core, highlighting the potential of their use to promote further π-conjugation and electronic delocalization. Likewise, the lower bandgap observed for o-SBN2 could be attributed to its higher aromatic content and molecular weight, elements that also contribute to more effective π-conjugation and greater electronic delocalization along the oligomeric chains [58,59].

In the o-SBB series, a slight bathochromic shift was observed for o-SBB2 (2.80 eV) as compared to o-SBB1 (2.82 eV). This behavior could be attributed to a more extensive π-conjugated system and the larger chain size of o-SBB2, which enhance electronic delocalization in the π-conjugated segments of binaphthyl-imine-TPS. These results are similar to those of previously reported silicon-based Schiff base oligomers and polymers, highlighting their influence in modulating the electronic properties of π-conjugated materials [34,50,60].

### 3.5. Computational Details of Oligo-SBs

**Figure 6:**
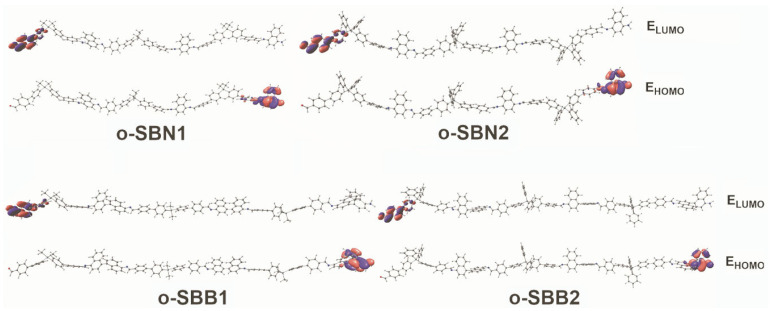
Frontier molecular orbitals for oligo-SBs as trimers.

To describe the geometrical parameters and the electronic and optical properties of all the synthesized materials, the trimeric structures of each oligomer were optimized and their TD-DFT computations were performed (Table 3). Although all simulated oligomers exhibited several torsions, these effects were compensated and resulted in a final linear structure, with minimal torsion in the optimized conformation for the local minimum (Figure 6).

The HOMO was located in all the oligomers studied over the amine-terminal region of the repetitive units, whereas the LUMO is located on the terminal aldehyde (Figure 6). In terms of energy, the o-SBN series exhibits a more unstable HOMO, while the localization of the LUMO is comparable among all the studied structures. This effect causes the naphthalene-based oligo-SBs derivatives to show a lower bandgap value. In every case, the HOMO is localized over the naphthalene-based region, while the LUMO is localized in the region of the silicon-based segments. In the case of o-SBB1, the LUMO is localized in a 1,1’-binaphtalene-based region.

To provide a rational explanation to the observed UV–vis spectra, TD-DFT calculations were performed over all the trimeric oligomers. As shown in Figures S4 and S5, a large number of molecular orbitals (MOs) are involved in each transition. Therefore, to provide a better description of these transitions, electron density difference map (EDDM) calculations were performed. This study shows the electronic localization before and after the electronic excitation. The involved MOs are localized in the same region of the oligomer; therefore, there is no charge displacement across the structure due to the excitation. Finally, as shown by the DFT results, these oligomers exhibit favorable properties as efficient charge transport and host materials, due to the difference in localization of the HOMO and LUMO orbitals and the calculated broad bandgap.

### 3.6. Comparative Analysis of Oligo-SBs with π-Conjugated Materials

The substitution of naphthalene units by 1,1’-binaphthalene moieties in DMS-containing oligomers revealed a significant decrease in the E_g_^opt^ value (ΔE = 0.85 eV, Table 2). This behavior was associated with a higher aromatic content, which enhanced the π-conjugation due to a significantly more coplanar conformation [50,58]. The same transformation in oligomers containing TPS cores resulted in a smaller effect (ΔE = 0.18 eV, Table 2).

The naphtalene-based decamer o-SBN2 exhibited the lowest E_g_^opt^ value (3.60 eV), which was attributed to the higher aromatic content and a structural design that promotes the coplanarity of the system, together with an increase in the molecular weight of this oligo-SB. This latter factor could enhance the bathochromic effect, similar to the previously reported π-conjugated oligo- and poly-SBs [51,61,62,63].

**Figure 7:**
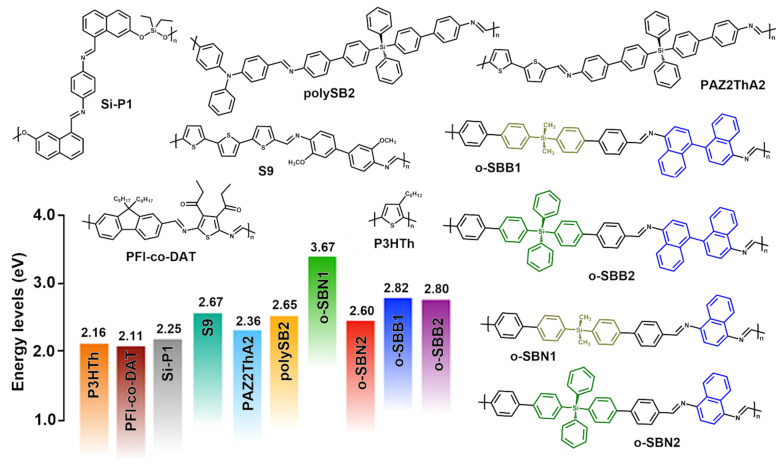
Bandgap energies of the prepared oligo-SBs compared to selected π-conjugated materials.

Likewise, the high aromatic content and the presence of DMS and TPS cores allows the development of new materials with wide bandgap values, along with adequate emission in the ultraviolet–visible region. Moreover, these materials exhibit high thermal stability, comparable with previously described π-conjugated polymers, both silicon-based and non-silicon-based (Figure 7) [41,49,64,65]. On the hand, the influence of silane cores on bandgap values indicates that their incorporation effectively modulates the thermal properties, allowing the design of materials with suitable optoelectronic properties [20,26,59].

As shown in Figure 7, the new Schiff base oligomers present bandgap values comparable to those of π-conjugated materials, which make them promising candidates for optoelectronic applications. Among these applications, it can cite their potential use as host materials or complementary layers in light-emitting devices [49,51,66,67]. In particular, o-SBN2, due to its suitable electronic, optical and thermal properties and its moderate solubility in CHCl_3_, could be considered as a promising host material for applications in light-emitting diodes or as a hole transport layer in optoelectronic devices. This proposal is supported by its bandgap value (2.60 eV), suitable for multicomponent systems in this type of application [8].

Finally, to improve the solubility of potential o-SBNs and o-SBBs derivatives, the incorporation of alkyl chains or alkoxy side groups as building block units could be used. This approach would improve solubility while maintaining suitable energy levels for applications in optoelectronic devices [8,48].

## 4. Conclusions

Four new silicon-based Schiff base oligomers, containing naphthalene (o-SBN1 and o-SBN2) and 1,1′-binaphthalene (o-SBB1 and o-SBB2) units, were synthesized from 1,4-diaminonaphthalene (dANP) and [1,1′-binaphthalene]-4,4′-diamine (dABN) with DMS- and TPS-based dialdehydes through high-temperature polycondensation reactions. The o-SBN and o-SBB series were obtained as oligomers of 5–10 repeating units with moderate molecular weights (5.10–11.53 kDa), as well as moderate-to-low solubility in common organic solvents, attributed to the high aromatic content and structural rigidity of the imine linkages in the backbone. Thermal analysis indicated that these oligo-SBs exhibit high thermal stability, with T5% values ranging from 458 °C to 530 °C, and relatively high T_g_ values, especially in the o-SBB series with a TPS core (above 340 °C). Optical and electronic results showed that both series exhibited absorption (298–387 nm) and emission (431–497 nm) bands in the ultraviolet–visible region. In particular, the naphthalene units of o-SBN2, combined with the higher molecular weight within the two series, promoted more effective π-conjugation. This effect was further reflected in the emission behavior of the oligo-SBs, which displayed strong emission and large Stokes shifts (126–195 nm). The bandgap values were observed to range from 2.60 eV to 3.67 eV, with the lowest value observed for o-SBN2, which could be associated with enhanced π-conjugation along the biphenyl-imine-naphthalene segments. This finding demonstrates that extension of the π-conjugated system enables the development of new materials with potential applications in optoelectronic devices. The optical and electronic properties of these oligo-SBs were compared and supported by computational simulations using DFT and TD-DFT methods in a dichloromethane solvent. These results showed that the electronic transitions are located over the same region of the modeled oligomer; thus, no large electronic delocalization is observed. Furthermore, a planar structure was shown with the obtained optimized structures.

## Figures and Tables

**Table 1:** GPC and thermal results of o-SBNs and o-SBBs.

Oligomer	M_w_ (kDa)	PDI ^a^	DP ^b^	T_5%_ (°C) ^c^	T_10%_ (°C) ^c^	R (%) ^d^	T_g_ (°C) ^e^
o-SBN1	5.10	1.78	5	458	481	63	136
o-SBN2	11.53	1.75	10	483	548	68	175
o-SBB1	n.d.	n.d.	-	504	526	66	347
o-SBB2	10.20	2.21	6	530	553	67	387

^a^ Polydispersity index. ^b^ Degree of polymerization. ^c^ Temperature at 5% and 10% weight loss, respectively. ^d^ Residual mass at 850 °C. ^e^ Glass transition temperature (second heating run). n.d.—not determined.

**Table 2:** Optical and electronic results of o-SBNs and o-SBBs in chloroform solutions.

Oligomer	λ_max_ (nm)	λ_max_^em^ (nm)	Stokes Shift (nm) ^a^	E_g_^opt^ (eV) ^b^
o-SBN1	298	431	133	3.67
o-SBN2	300, 387 ^c^	488	101	2.60
o-SBB1	302	497	195	2.82
o-SBB2	300, 369 ^c^	495	106	2.80

^a^ λ_max_^em^-λ_max_^abs^. ^b^ 1241/λ _onset_^abs^. ^c^ Excitation wavenumber used for PL analysis.

**Table 3:** Computed wavenumber values (λ_Th_), oscillator strengths (*f*), contributing FMOs and their respective percentage contributions (*p*) for the investigated oligo-SBs in dichloromethane.

Oligomer	λ_Th_ (nm)	ƒ	Composition	*p* (%)	E_HOMO_ (eV)	E_LUMO_ (eV)	E_g_ (eV)
**o-SBN1**	298	0.8952	HOMO-4 → LUMO+3	22	−4.99	−2.1	2.89
			HOMO-1 → LUMO+6	11			
			HOMO-5 → LUMO+2	9			
			HOMO-4 → LUMO+2	9			
			HOMO-1 → LUMO+13	7			
			HOMO-3 → LUMO+5	6			
			HOMO-1 → LUMO+16	5			
**o-SBN2**	327	0.6502	HOMO-1 → LUMO+7	52	−4.87	−1.96	2.91
			HOMO-1 → LUMO+6	17			
			HOMO-1 → LUMO+17	8			
			HOMO-4 → LUMO+2	5			
	303	0.7602	HOMO-2 → LUMO+6	23			
			HOMO-6 → LUMO+1	21			
			HOMO-5 → LUMO+1	10			
			HOMO-11 → LUMO+1	9			
			HOMO-2 → LUMO+7	6			
			HOMO-3 → LUMO+2	5			
**o-SBB1**	326	0.5738	HOMO-2 → LUMO+6	81	−5.13	−1.97	3.17
			HOMO-2 → LUMO+8	8			
			HOMO-4 → LUMO+8	70			
			HOMO-6 → LUMO+1	15			
			HOMO-4 → LUMO+6	9			
	310	1.2735	HOMO-4 → LUMO+8	70			
			HOMO-6 → LUMO+1	15			
			HOMO-4 → LUMO+6	9			
**o-SBB2**	397	0.1698	HOMO-5 → LUMO+4	58	−5.11	−1.97	3.14
			HOMO-2 → LUMO+4	28			
			HOMO-2 → LUMO+2	6			
			HOMO-4 → LUMO+9	34			
			HOMO-3 → LUMO+9	23			
			HOMO-4 → LUMO+10	7			
			HOMO-6 → LUMO+1	6			
			HOMO-3 → LUMO+10	5			
	313	0.8675	HOMO-4 → LUMO+9	34			
			HOMO-3 → LUMO+9	23			
			HOMO-4 → LUMO+10	7			
			HOMO-6 → LUMO+1	6			
			HOMO-3 → LUMO+10	5			

## Data Availability

Data are contained within the article and Supplementary Materials.

## Supplementary Materials

The following supporting information can be downloaded at: https://www.mdpi.com/article/10.3390/polym17101316/s1, Figure S1: Synthesis of [1,1′-binaphthalene]-4,4′-diamine (dABN) and silicon-containing dialdehyde monomers (M1 and M2). Table S1: Solubility test results of o-SBNs and o-BBs. Figure S2: GPC traces for o-SBNs and o-SBB2. Figure S3: DSC curves of o-SBNs and o-SBBs in nitrogen atmosphere. (a) o-SBN1, (b) o-SBN2, (c) o-SBB1 and (d) o-SBB2. Figure S4: EDDM isosurfaces for the naphthalene-based oligo-SBs. (a) o-SBN1 at 298 nm, (b) o-SBN2 at 327 nm and 303 nm. Figure S5: EDDM isosurfaces for the 1,1′-binaphthalene-based oligo-SBs. (a) o-SBB1 at 327 nm and 310 nm, (b) o-SBB2 at 397 nm and 313 nm. Figure S6: NMR (CDCl_3_) spectra for M1. a) ^1^H, ^13^C and c) ^29^Si. Figure S7: NMR (CDCl_3_) spectra for M2. a) ^1^H, ^13^C and c) ^29^Si.
