# Supplementary material for: Silicon-Containing π-Conjugated Schiff Base Oligomers with Naphthalene or Binaphthalene Moieties in the Backbone: Synthesis and Study of Properties
Source: Polymers (Basel). 2025 May 12;17(10):1316. doi: 10.3390/polym17101316 (PMC12115331; doi:10.3390/polym17101316)
Supplement: Supplementary file 1 [file polymers-17-01316-s001.zip › polymers-3582837-supplementary.pdf]

Supporting Information

Silicon-containing π-conjugated Schiff base oligomers with naphthalene or binaphthalene moieties in the backbone. Synthesis and study of properties.

Enzo González[a], Alexis F. González[b], Andrea P. Mariman[b], Camilo I. Jara[c], Joel D. Velázquez[c], César Saldías[b], Eduardo Schott[d], Ximena Zarate[e], Alain Tundidor-Camba[f], Patricio A. Sobarzo[g]*, Claudio A. Terraza[a]*

[a] Research Laboratory for Organic Polymers, Faculty of Chemistry and of Pharmacy, Pontificia Universidad Católica de Chile, P.O. Box. 306, Post 22, Santiago, Chile.
[b] Department of Physical Chemistry, Pontificia Universidad Católica de Chile, Santiago, Chile
[c] Instituto de Ciencias Químicas, Facultad de Ciencias, Universidad Austral de Chile, Valdivia, Chile
[d] Department of Inorganic Chemistry, Pontificia Universidad Católica de Chile, Santiago, Chile
[e] Instituto de Ciencias Químicas Aplicadas, Facultad de Ingeniería, Universidad Autónoma de Chile, Santiago, Chile
[f] Department of Chemical & Biological Engineering, The University of Alabama, Alabama, United States
[g] Departamento de Polímeros, Facultad de Ciencias Químicas, Universidad de Concepción, Edmundo Larenas 129, Concepción, Casilla 160-C.

*Corresponding authors: Patricio A. Sobarzo (patriciosobarzo@udec.cl), Claudio A. Terraza (cterraza@uc.cl)

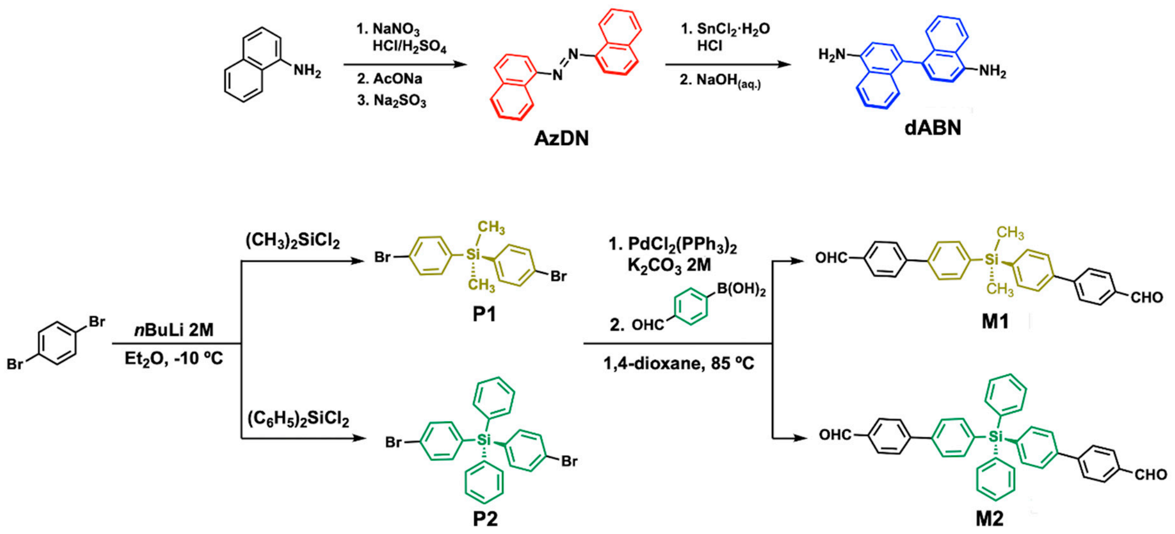

Figure S1. Synthesis of [1,1'-binaphthalene]-4,4'-diamine (dABN) and silicon-containing dialdehyde monomers (M1 and M2).

Table S1. Solubility test results of o-SBNs and o-BBs.

Solvent	o-SBN1	o-SBN2	o-SBB1	o-SSB2
Alcohols (C_{1-4})	I	I	I	I
DCM	I	I	PS[b]	I
$CHCl_3$	PS[a]	PS[b]	S	PS[a]
ACN	I	I	I	PS[b]
Acetone	I	I	I	PS[b]
THF	PS[b]	PI[b]	S	PS[b]
Chlorobenzene	I	I	PS[a]	PS[b]
DMF	PS[b]	I	PS[b]	PS[a]
DMSO	PS[b]	PS[b]	PS[b]	PS[a]

Solubility measured at RT and at the boiling point of the solvent or 60 °C. S: Soluble, PS: Partially soluble, I: Insoluble. [a] Swelling is observed in the undissolved part, which does not dissolve with heat. [b] Most of the sample remains undissolved at RT, increasing in solubility with heat.

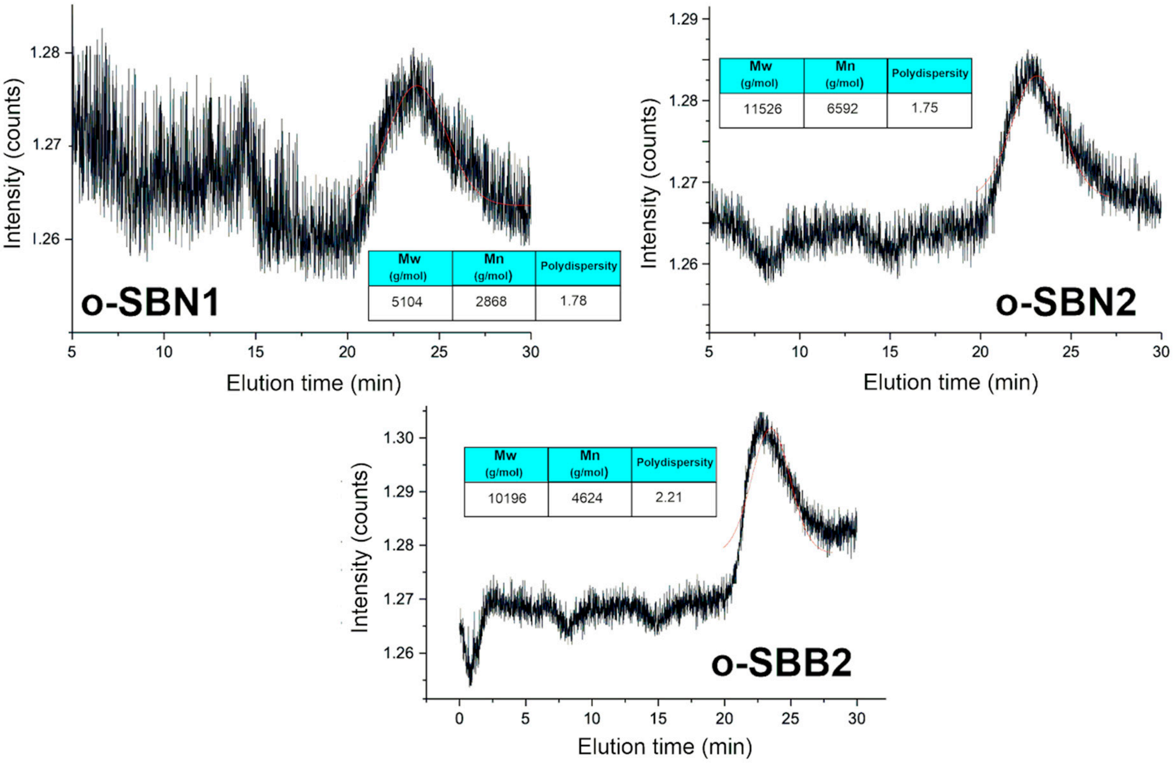

Figure S2. GPC traces for o-SBNs and o-SBB2.

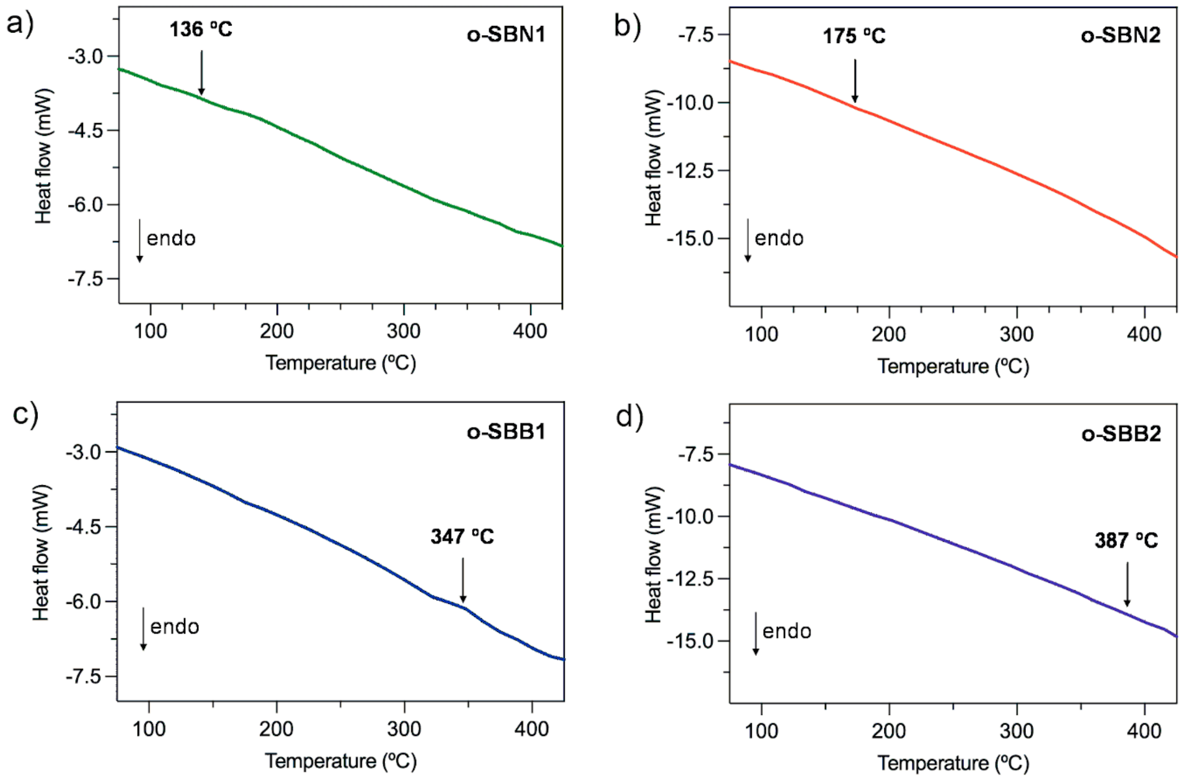

Figure S3. DSC curves of o-SBNs and o-SBBs in nitrogen atmosphere. a) **o-SBN1**, b) **o-SBN2**, c) **o-SBB1** and d) **o-SBB2**.

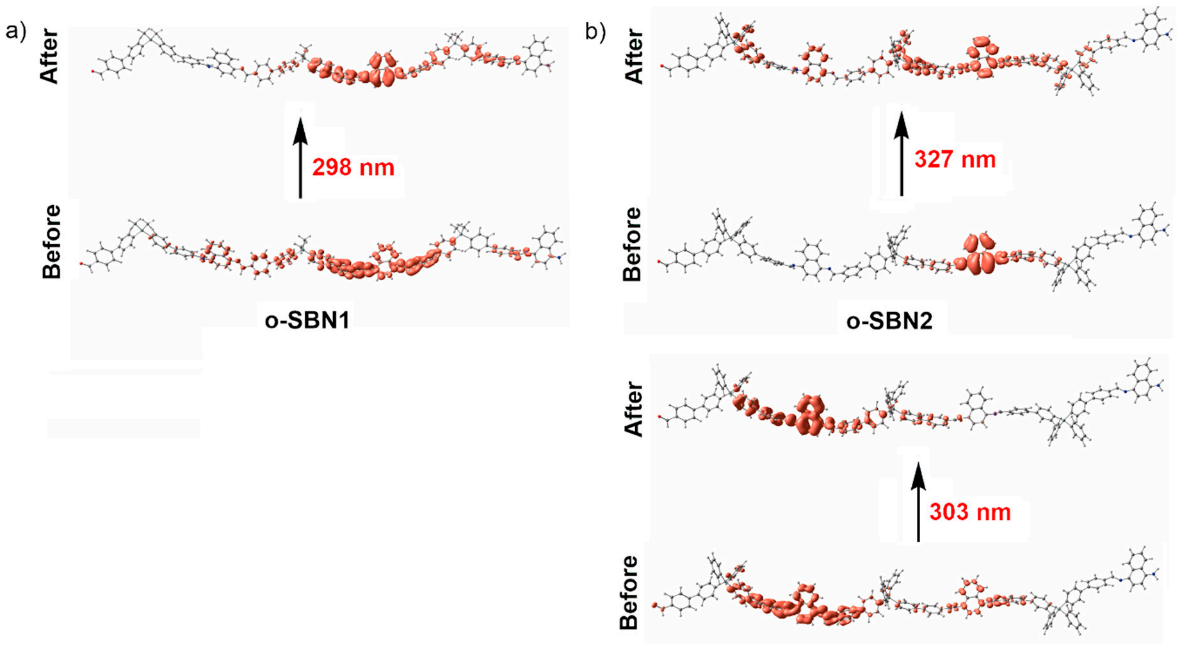

Figure S4. EDDM isosurfaces for the naphthalene-based oligo-SBs. (a) o-SBN1 at 298 nm, (b) o-SBN2 at 327 nm and 303 nm.

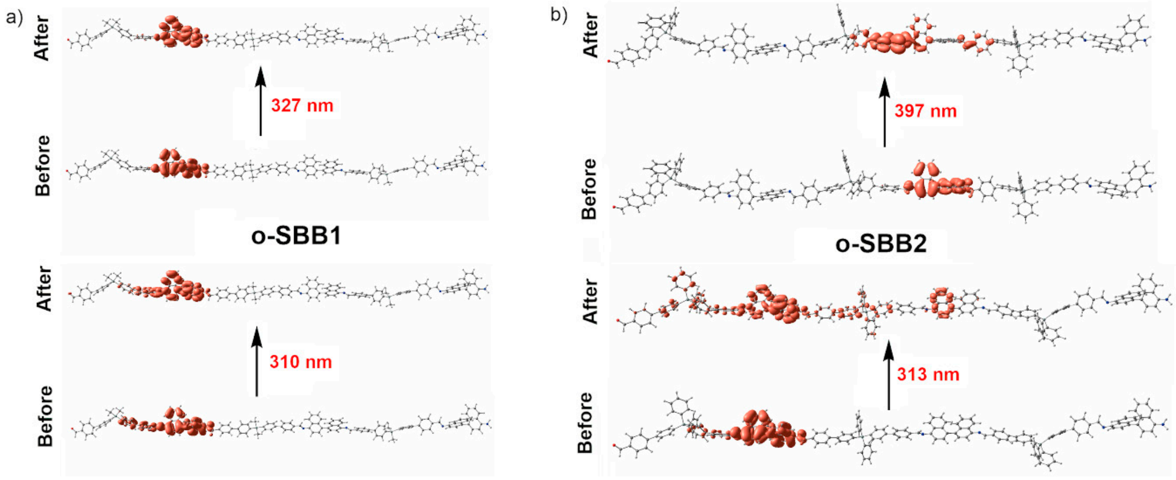

Figure S5. EDDM isosurfaces for the 1,1′-binaphthalene-based oligo-SBs. (a) o-SBB1 at 327 nm and 310 nm, (b) o-SBB2 at 397 nm and 313 nm.

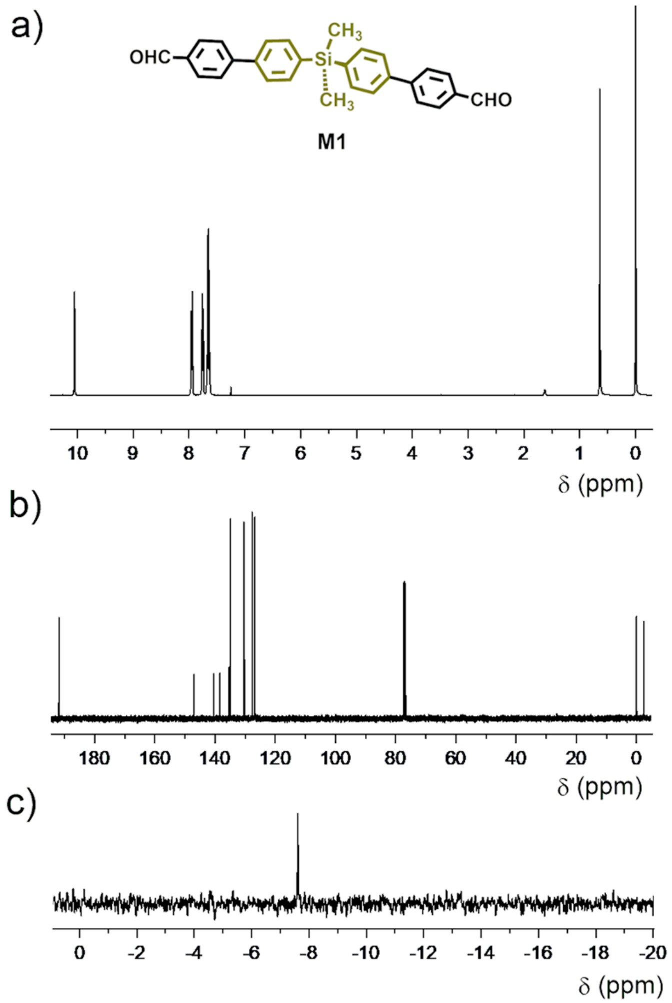

Figure S6. NMR ($CDCl_3$) spectra for **M1**. a) ^{1}H, ^{13}C and c) ^{29}Si.

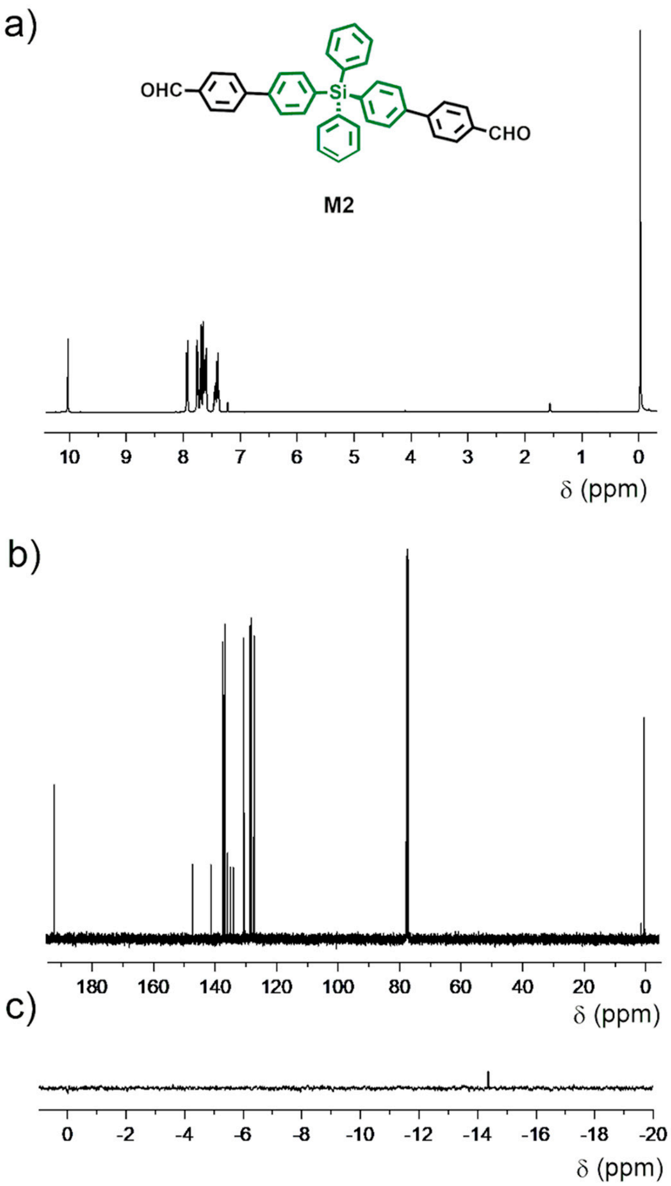

Figure S7. NMR ($CDCl_3$) spectra for **M2**. a) ^1H, ^{13}C and c) ^{29}Si.
